# HIV-1 reverse transcriptase mutations that confer decreased in vitro susceptibility to anti-RT DNA aptamer RT1t49 confer cross resistance to other anti-RT aptamers but not to standard RT inhibitors

**DOI:** 10.1186/1742-6405-2-8

**Published:** 2005-10-05

**Authors:** Timothy S Fisher, Pheroze Joshi, Vinayaka R Prasad

**Affiliations:** 1 Department of Microbiology and Immunology, Albert Einstein College of Medicine, Bronx, New York, USA; 2 Division of Cardiovascular Diseases, Merck Research Laboratories, Rahway, New Jersey 07065, USA

## Abstract

RNA and DNA aptamers specific for HIV-1 reverse transcriptase (RT) can inhibit reverse transcription *in vitro*. RNA aptamers have been shown to potently block HIV-1 replication in culture. We previously reported mutants of HIV-1 RT with substitutions N255D or N265D that display resistance to the DNA aptamer RT1t49. Variant viruses bearing these mutations singly or in combination were compromised for replication. In order to address the wider applicability of such aptamers, HIV-1 RT variants containing the N255D, N265D or both (Dbl) were tested for the extent of their cross-resistance to other DNA/RNA aptamers as well as to other RT inhibitors. Both N265D and Dbl RTs were resistant to most aptamers tested. N255D mutant displayed mild resistance to two of the DNA aptamers, little change in sensitivity to three and hypersensitivity to one. Although all mutants displayed wild type-like ribonuclease H activity, their activity was compromised under conditions that prevent re-binding. This suggests that the processivity defect caused by these mutations can also affect RNase H function thus contributing further to the replication defect in mutant viruses. These results indicate that mutants conferring resistance to anti-RT aptamers significantly affect many HIV-1 RT enzymatic activities, which could contribute to preventing the development of resistance in vivo. If such mutations were to arise in vivo, our results suggest that variant viruses should remain susceptible to many existing anti-RT inhibitors. This result was tempered by the observation that NRTI-resistance mutations such as K65R can confer resistance to some anti-RT aptamers.

## Background

The reverse transcriptase (RT) of the human immunodeficiency virus type 1 (HIV-1) is a multifunctional enzyme, capable of several discrete activities required for viral replication [1]. These essential activities include DNA- and RNA-dependent DNA polymerase (DDDP and RDDP), ribonuclease H (RNase H), strand transfer and strand displacement activities. Native HIV-1 RT is a heterodimer of p66 and p51 subunits, of which the p66 subunit contains both the polymerase and RNase H domains. The p51 subunit is derived by proteolytic cleavage of the p66 subunit and is thought to play both an architectural role in the context of the p66/p51 heterodimer as well as facilitate template·primer binding [2].

Due to its essential role in synthesizing the double-stranded proviral DNA from single-stranded HIV-1 RNA genome, the HIV-1 RT is a major target of current antiviral therapies directed against HIV-1. Current anti-HIV drug regimens, termed highly active antiretroviral therapy (HAART), typically consist of a combination of at least three antiretroviral drugs, with two or more nucleotide reverse transcriptase inhibitors (NRTIs) being a staple of most regimens [3,4]. In addition to NRTIs, which are both competitive inhibitors and chain-terminators, the non-nucleoside reverse transcriptase inhibitors (NNRTIs) consist of structurally dissimilar hydrophobic compounds that bind to a hydrophobic pocket on the RT adjacent to, but distinct from, the active site, which accommodates dNTPs and NRTIs. While HAART regimens have decreased both the mortality and morbidity of HIV-infected individuals, several factors contribute to drug failure. The highly error-prone nature of HIV-1 RT [5,6] combined with a robust rate of viral replication [7,8] provides the virus with an ideal context for the emergence of resistant variants. In addition, the significant toxicity associated with the current crop of anti-HIV drugs often leads to noncompliance, which in turn results in treatment failure [9]. For these reasons, there is a high level of interest in the development of more potent anti-HIV inhibitors that are both less likely to lead to drug-resistant variants and display less toxicity in patients.

Among a number of anti-HIV agents being developed for potential use in the treatment of AIDS are nucleic acid-based inhibitors that can serve as useful complementary therapies [10]. Of these, three nucleic acid-based approaches have recently been shown to have potent influence on HIV replication. In one, using a long antisense *env *RNA approach, strong inhibition of HIV replication was observed in cultured T cells [11]. This approach combined with a lentiviral vector completed the phase I clinical trials and is about to enter phase II trials [12]. The second approach, RNA interference (RNAi), uses a natural cellular pathway for gene silencing via small interfering RNAs [13-16]. The third approach is based on DNA and RNA aptamers that are derived by the iterative process of SELEX, to bind to specific protein targets [17] and has been recently shown to be effective in blocking HIV replication [18-20].

Tuerk and Gold first reported the isolation of RNA aptamers targeting HIV-1 RT using an iterative selection process of binding, washing and eluting the RNAs from a random library of RNA sequences [21]. Subsequent reports showed that both DNA and RNA aptamers generated against HIV-1 RT [22,23] are highly specific (do not bind to FIV or MuLV RTs), bind tightly to HIV-1 RT (Kd in the range of 0.05 to 50 nM) and competitively inhibit its polymerase activity. The crystal structure of an HIV-1 RT complexed with an anti-RT aptamer confirmed that the aptamer RNA is bound by the template·primer cleft of HIV RT [24]. Since these aptamers compete with template·primer for the template-binding cleft, they have been termed template analog RT inhibitors (TRTIs) [25]. In order to test the utility of anti-RT aptamers as inhibitors of HIV replication, we previously expressed RNA aptamers specific to HIV-1 RT in Jurkat T cells and showed that the tightest binding aptamers were able to potently block the infection and the subsequent spread of HIV-1 in cell culture [19]. In addition, five of the nine different clades of HIV-1 tested and all of the RTI and PI-resistant isolates tested were also severely inhibited [19]. The block was found to be in the early steps of reverse transcription. A subsequent report, using single cycle infection experiments involving one RNA aptamer (1.1), has confirmed the strong inhibition of HIV-1 replication by anti-RT aptamers [18].

It has been suggested that resistance to aptamers in vivo may be difficult due to the presumed need for multiple mutations required to disengage the interactions via the large interface between the inhibitor and HIV-1 RT [19]. In order to address this notion, we previously used a phenotypic screen based on the in situ detection of RNA-dependent DNA polymerase activity of HIV-1 RT expressed within bacterial colonies, and isolated two variants of recombinant HIV-1 RT bearing the substitutions N255D or N265D, both of which displayed in vitro resistance to the DNA aptamer RT1t49 [25]. The mechanism of resistance to these aptamers appeared to be based on the loss of affinity to the aptamer and the level of resistance increased from a range of 2- to 11-fold for single mutations to ~150-fold when the two mutations were combined. When the mutant RT sequences were incorporated into molecular clones of HIV-1, the resulting HIV virions were compromised for infectivity in single cycle infection assays and for virus replication in multi-day cell culture replication experiments [25]. Thus, despite the biochemically robust enzymatic activity that allows one to measure drug-susceptibility levels of the mutant RTs, it appeared that the aptamer-resistance mutations tend to target biologically crucial sites. In support of this view, we have further demonstrated that all three mutants (the N255D, N265D and the double mutant (Dbl) RTs containing both mutations) are defective for processive DNA-dependent DNA polymerase activity (DDDP), although N265D retained processive polymerization activity on RNA templates [26].

The data available demonstrate the utility of aptamers in inhibiting HIV-1 replication. In addition to their exquisite specificity, high level of resistance to anti-RT aptamers appears to require multiple mutations, which affect the polymerase activity of the enzyme. Although resistant virus particles could be produced from molecular clones with mutant RTs, the mutant viruses displayed reduced replication competence and thus lacked a competitive edge in the presence of a large complexity of virus population. It is important to know whether the aptamer-resistant RTs retain their sensitivity to other classes of anti-RT drugs. In the present communication, we have further evaluated the enzymatic properties of the aptamer-resistant RTs. First, we measured the breadth of cross-resistance to other anti-RT inhibitors, including several standard NRTIs and NNRTIs and otherDNA and RNA aptamers specific to HIV-1 RT. Second, we have investigated biochemical defects that may be responsible for their reduced replication fitness. These are important questions concerning the potential of anti-RT aptamers as a viable treatment option. We find that these mutants are resistant to several additional DNA aptamers, thus suggesting a common contact point on HIV-1 RT to this new class of nucleic acid-based anti-RT inhibitors. Importantly, we find that the aptamer-resistant mutations retain wild-type susceptibilities to all NRTIs and NNRTIs tested. Furthermore, amongst a series of NRTI-resistant HIV-1 RT variants, only the K65R RT mutant displayed a significant (5-fold) level of resistance to RT1t49. Our results, combined with previous reports, demonstrate that mutations conferring resistance to the DNA aptamer, RT1t49 *in vitro *affect the RNase H domain in addition to previously shown effect on polymerase domain, both of which are essential for efficient viral DNA replication.

## Results

### Cross-resistance of DNA aptamer RT1t49-resistant mutants of HIV-1 RT to other inhibitors

We investigated whether the aptamer-resistance mutations, N255D and N265D, would affect the sensitivity of HIV-1 RT to other DNA and RNA aptamers directed to HIV-1 RT [21,23]. RT1t49 and 5 other DNA aptamers representing each of the six classes of DNA aptamers described by Schneider et al. [23] and a single RNA aptamer 1.1 (termed Rknot 1.1 here) were selected. Using a steady-state nucleotide incorporation assay, a similar pattern of resistance to that of RT1t49 was observed with DNA aptamers RT26, RT4, and RT6 (Table 1). In each of these cases, the N265D mutation conferred a greater degree of resistance compared to the N255D mutation. In addition, the presence of both mutations led to an even greater degree of resistance (6- to 27-fold) to aptamers in this group. In contrast, both N255D and Dbl mutant RTs were hypersensitive (10-fold) to DNA aptamer RT8, while the N265D mutant displayed wild type levels of sensitivity (Table 1). However, with respect to the DNA aptamer RT10 and the single RNA aptamer tested (Rknot1.1), the N255D mutant was similar to wild type, while both N265D and Dbl mutants were significantly resistant. The similarity between resistance profiles of N255D and N265D mutant RTs to both DNA aptamers (RT1t49, RT26, RT4, RT6) suggest that the residues N255 and N265 are important contacts for several classes of DNA aptamers.

**Table 1:** Resistance of Purified RTs to DNA and RNA Aptamers. Assays were performed as described previously [34]. Data represent mean ± SEM of three independent experiments.

	WT		N255D		N265D		Dbl	
^a^TRTI	^b^IC_50_, nM	^c^Ratio	IC_50_, nM	Ratio	IC_50_, nM	Ratio	IC_50_, nM	Ratio
RT1t49^d ^ RT26^f^	1.6 4.0 ± 0.05	1 1	7.9 7.6 ± 0.1	4.9 1.9	17.4 11.2 ± 0.1	10.9 2.8	245 24 ± 0.1	153 6
RT4^f^	38 ± 1.2	1	80 ± 3.7	2.1	1015 ± 16	27	> 1000	> 27^e^
RT6^f^	19.6 ± 0.1	1	26 ± 0.7	1.3	87 ± 2.2	4.4	142 ± 3.4	7.2
RT8^f^	19.5 ± 0.3	1	2.0 ± 0.02	0.1	17.2 ± 0.2	0.9	3.0 ± 0.02	0.1
RT10^f^	82 ± 2.5	1	57 ± 1.4	0.7	923 ± 8.9	11	509 ± 4.2	6
Rknot 1.1^f^	1.4 ± 0.02	1	0.8 ± 0.01	0.8	2.5 ± 0.04	2	4.5 ± 0.08	4

^a^Described in references 23 and 25.

^b^Concentration of aptamer at which 50% of the activity was inhibited.

^c^Fold increase or decrease over the IC_50 _for the wild type (WT) RT.

^d^Reproduced from Fisher et al. [25] ^e^At the highest concentration of aptamer tested (1000 nM), the Dbl mutant retained 70% of its activity, thus the actual IC_50 _would be much higher.

^f^Aptamer sequences: RT1t49: 5' ATCCGCCTGATTAGCGATACTCAGAAGGATAAACTGTCCAGAACTTGGA3'

RT26: 5'ATCCGCCTGATTAGCGATACTTACGTGAGCGTGCTGTCCCCTAAAGGTGATACGTCACTTGAGCAAAATC ACCTGCAGGGG3'

RT4:5'ATCCGCCTGATTAGCGATACTTTAGCAAAGTTGAAGCCGGACTAACAAGCTCTACGACTTGAGCAAAATCA CCTGCAGGGG3'

RT6: 5'ATCCGCCTGATTAGCGATACTCAGGCGTTAGGGAAGGGCGTCGAAAGCAGGGTGGGACTTGAGCAAAATCA CCTGAGGGG3'

RT8:5'ATCCGCCTGATTAGCGATACTAGCCAGTCAAGTTAATGGGTGCCATGCAGAAGCAACTTGAGCAAAATCA CCTGCAGGGG3'

RT10:5'ATCCGCCTGATTAGCGATACTTATTTGCCCCTGCAGGCCGCAGGAGTGCAGCAGTACTTGAGCAAAATCA CCTGCAGGGG3'

Rknot 1.1: 5'GGGAGAUUCCGUUUUCAGUCGGGAAAAACUGAA3'

We next tested cross-resistance of these variant RTs to conventional RT inhibitors such as NRTIs and NNRTIs. Each of the single mutants, N255D and N265D, and the double mutant RTs were tested for their sensitivity to a selected set of NRTIs (AZTTP, ddATP, ddCTP, d4TTP and 3TCTP) or the NNRTIs (nevirapine and delavirdine). Interestingly, neither the single mutations nor the double mutants altered the susceptibility of HIV-1 RT to any of these RT inhibitors (Table 2).

**Table 2:** Sensitivity of aptamer-resistant RTs to NRTIs and NNRTIsAssays were performed as described in the text. Data represent mean ± SEM of three independent experiments.

	WT		N255D		N265D		Dbl	
Inhibitor	^a^IC_50_, μM	^b^Ratio	IC_50_, μM	Ratio	IC_50_, μM	Ratio	IC_50_, μM	Ratio
AZTTP	1.83 ± 0.25	1	2.67 ± 0.09	1.45	1.74 ± 0.28	0.9	2.43 ± 0.26	1.3
ddATP	0.93 ± 0.18	1	1.07 ± 0.11	1.2	0.84 ± 0.04	0.9	0.91 ± 0.07	1
ddCTP	0.88 ± 0.20	1	0.69 ± 0.07	0.8	0.72 ± 0.17	0.8	0.96 ± 0.09	1.1
3TCTP	4.37 ± 0.87	1	2.51 ± 1.04	0.6	5.02 ± 1.22	1.1	2.69 ± 0.95	0.6
d4TTP	0.79 ± 0.05	1	0.83 ± 0.14	1	0.64 ± 0.12	0.8	0.91 ± 0.10	1.2
Nevirapine	0.10 ± 0.01	1	0.06 ± 0.02	0.6	0.09 ± 0.03	0.9	0.07 ± 0.01	0.7
Delavirdine	0.37 ± 0.02	1	0.64 ± 0.03	1.7	0.36 ± 0.01	1	0.31 ± 0.01	1

^a^Concentration of inhibitor at which 50% of the activity was inhibited.

^b^Ratio of this enzyme's drug susceptibility to that of wild type.

### Some NRTI-resistant RTs display low-level resistance to the DNA aptamer, RT1t49

Similar experiments were performed to determine the effectiveness of the DNA aptamer, RT1t49 in inhibiting the polymerase activities of several NRTI-resistant mutants of HIV-1 RT. Variants of HIV-1 RT shown to confer resistance to AZT (T215Y/M41L) and ddI and ddC (L74V) were sensitive to inhibition by RT1t49 (Table 3). In contrast, mutations shown to confer resistance to multiple NRTIs, including E89G, K65R and M184V displayed low levels of resistance to RT1t49 (2–5 fold), with K65R displaying the highest level of resistance (5-fold). K65R is known to cause resistance to all clinically approved NRTIs except AZT in patients. However, in vitro biochemical experiments do show some resistance to AZTTP and it has been suggested this is due to K65R decreasing the rate of AZTMP excision. The residues E89 and K65 are located in template grip region of palm and the β3-β4 hairpin loop of fingers regions respectively. Both these regions are known to contact different parts of the template·primer molecule. Thus, these results suggest that the RT1t49 aptamer may make contact with several of the key regions of RT involved in template·primer contact.

**Table 3:** Sensitivity of NRTI-resistant RTs to the DNA aptamer RT1t49Assays were performed as described previously [34]. Data represent mean ± SEM of three independent experiments.

Enzyme	IC_50_, nM	Ratio
WT	1.5 ± 0.03	1
E89G	4.9 ± 0.06	3.3
K65R	8.0 ± 0.05	5.3
L74V	0.86 ± 0.02	0.6
M184V	3.2 ± 0.05	2.1
T215Y/M41L	2.1 ± 0.04	1.4

^a^Concentration of inhibitor at which 50% of the activity was inhibited over the IC_50 _for wild type (WT) RT

### Anti-HIV RT aptamer-resistant RT mutants are defective for RNase H-mediated cleavage

**Figure 1:**
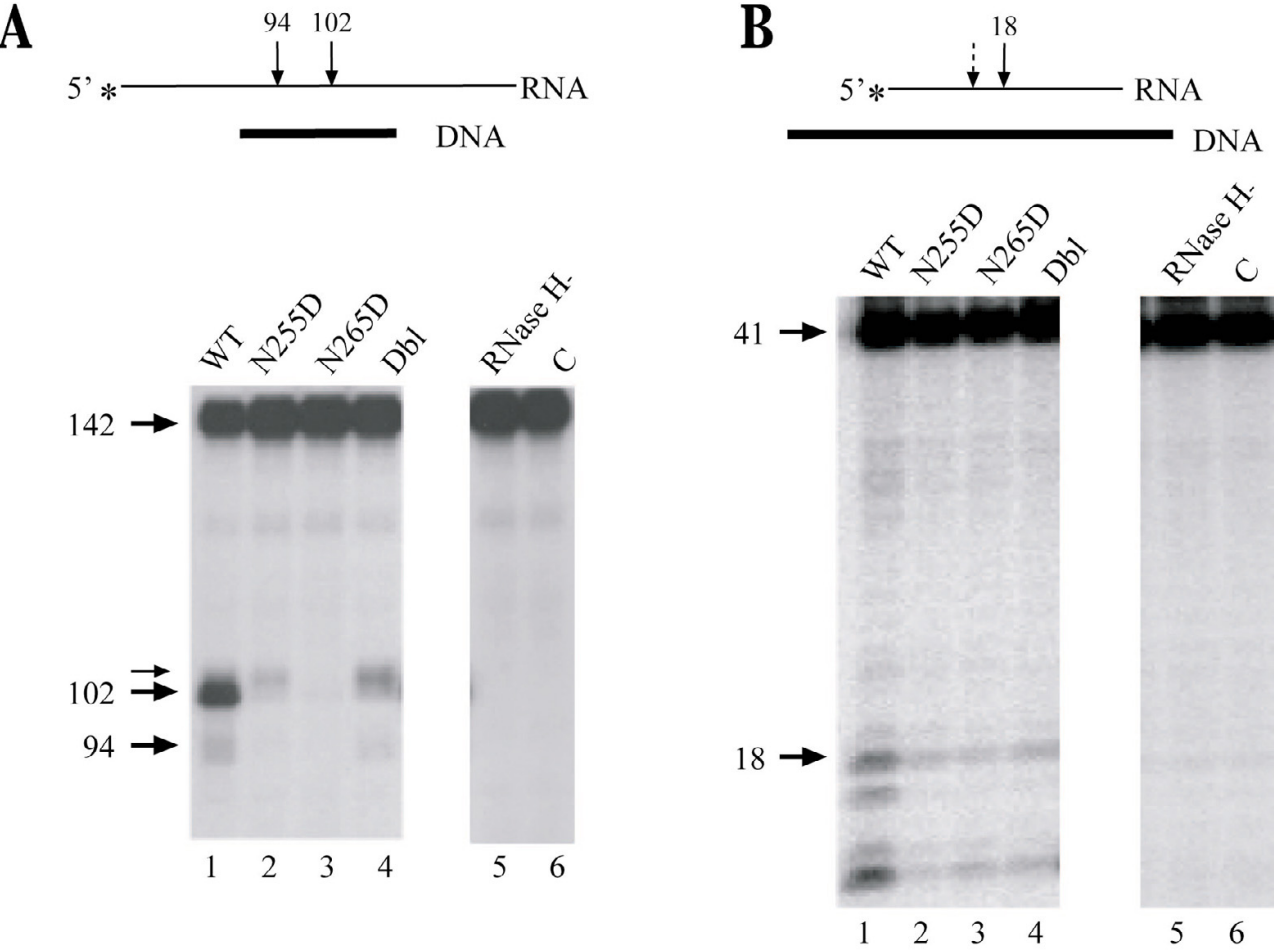
**RNase H cleavage of RNA.DNA hybrids by wild type (WT) and mutant RTs in the presence of a heparin challenge. **A. Polymerase-dependent RNase H clevage. The substrate, as diagrammed at the top, consisted of a 142nt heteropolymeric RNA (thin line) annealed to a 30nt DNA primer (thick line). Arrows indicate the expected sites of cleavage. Reactions were performed in the absence of dNTPs and in the presence of a heparin trap. Control reactions were performed in which either no enzyme was added (C), or an RNAse H-defective mutant (E478Q) was added (RNase H^-^) (see Methods secion). Cleavage products were resolved on a denaturing 6% polyacrylamide gel. The sizes of the resultant radiolabeled products are represented to the left of the gel panels (including a minor product). B. RNA 5'-end-directed RNase H cleavage. The substrate was a 41nt heteropolymeric RNA annealed to a 47nt DNA template. Reaction conditions were otherwise identical to those in panel A, and are described under 'Materials and Methods' section. Cleavage products were resolved on a denaturing 12% polyacrylamide gel. The sizes of the resultant radiolabeled products are represented to the left of the gel panels.

We next tested the impact of aptamer resistance mutations on RNase H activity associated with HIV-1 RT. Previous studies have shown that alanine substitutions at several residues within the minor groove binding track (MGBT) [27] affect not only RT processivity, but also the specificity of RNase H-catalyzed removal of the polypurine tract (PPT) primer [28]. Both N255 and N265 are located in the α H helix of HIV-1 RT, and are therefore in close proximity to the MGBT. Both the polymerase-dependent and RNA 5'-end-directed RNase H activity of wild type and aptamer-resistant RTs were tested. Under conditions that prevent the RT from rebinding the substrate RNA.DNA duplex, the aptamer-resistant RTs were found to be deficient in both polymerase-dependent and RNA 5'-end-directed RNase H activities (Figure 1A and 1B). In this case, RT was pre-bound to the DNA.RNA substrate before reactions were initiated by adding both MgCl_2 _and heparin as a competitive trap. Therefore any cleavage products formed were the result of a single binding event.

Polymerase-dependent RNase H cleavage by wild type RT results in the formation of a 102-nt product (Figure 1A, lane 1). The smaller 94-nt product is the result of subsequent 3' → 5' directional nucleolytic activity of HIV-1 RT RNase H [29,30]. Under identical conditions, each of the aptamer-resistant RTs failed to produce significant amounts of either 102-nt or 94-nt products (Figure 1A, lanes 2–4). While there appeared to be a limited cleavage by both N255D and Dbl mutants, products formed were altered in size compared to wild type products (Figure 1A, lane 1 vs. lanes 2 and 4). These results indicate that although the N255D and Dbl mutant RTs possess residual polymerase-dependent RNase H activity under single cycle cleavage conditions, the specificity of cleavage under such conditions has not been retained.

Similar reactions were carried out to determine the effect of aptamer resistance mutations on HIV-1 RT RNA 5'-end-directed RNase H activity (Figure 1B). Following completion of minus strand DNA synthesis, RNA fragments left behind are removed by this activity in order to facilitate plus strand DNA synthesis. Both wild type and aptamer resistant RTs were incubated with the RNA:DNA substrate before reactions were initiated by adding MgCl_2 _and heparin trap. Wild type RT efficiently cleaved the RNA:DNA substrate, resulting in the expected 18-nt cleavage product in addition to several smaller products that are the result of processive cleavage. In contrast, reactions in which aptamer-resistant RTs were included resulted in minimal cleavage products (Figure 1B, lanes 2–4). Together, these results indicate that both aptamer resistance mutations N255D and N265D result in a severe reduction of HIV-1 RT mediated RNase H cleavage under challenged conditions.

**Figure 2:**
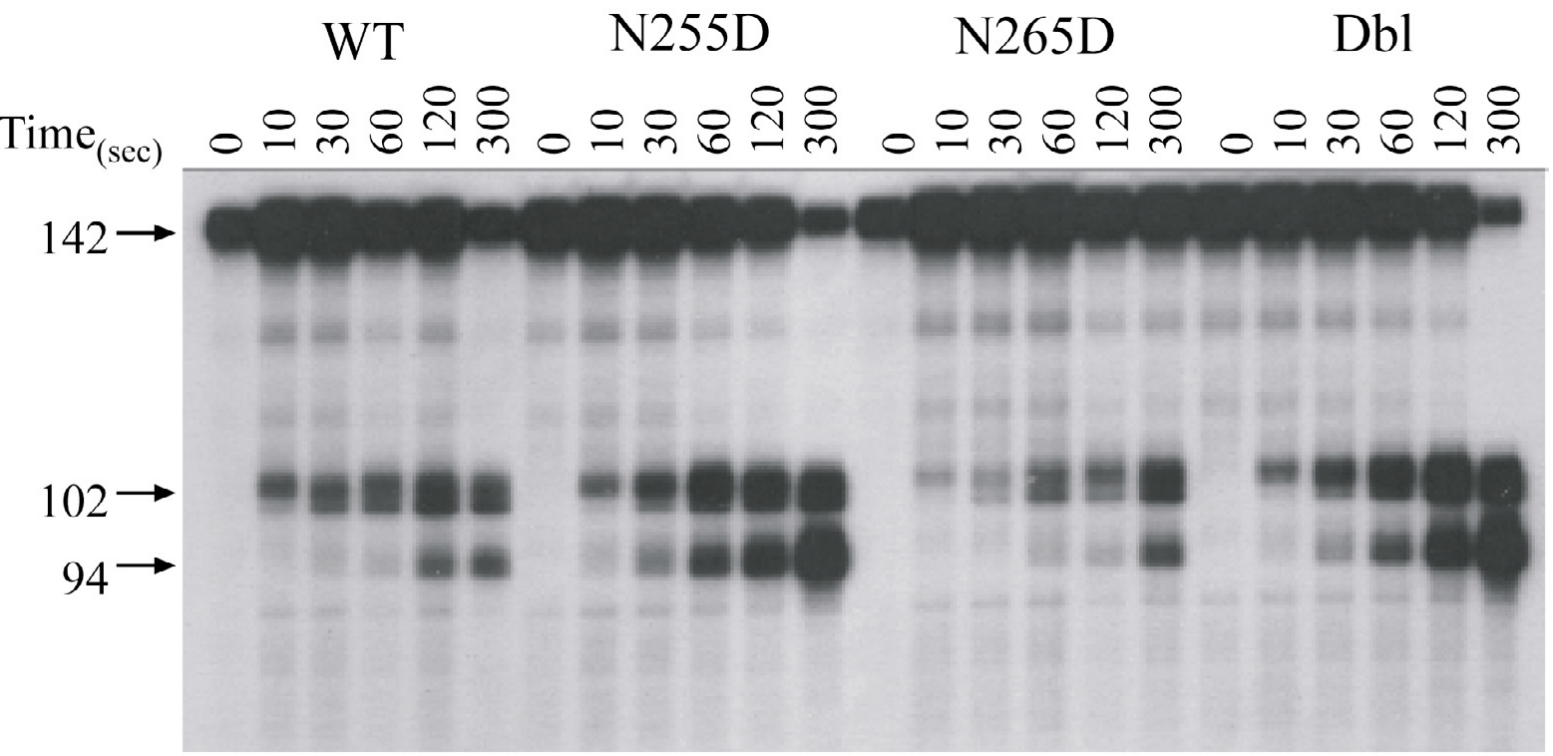
**Comparison of polymerase-dependent RNase H activities of wild type (WT), and mutant RTs. **HIV-1 RT and template·primer substrates were combined and time course reactions were performed with a 5'-end labeled 142nt RNA template and 30nt DNA primer for 0, 10, 30, 60, 120 and 300 seconds. Cleavage products were resolved by denaturing 6%. The product sizes are indicated to the left of the panel.

The observed defect in Figure 1 appears to be due to a loss in substrate affinity and not due to defect in the RNase H catalytic activity of these mutant RTs. To determine whether these mutant RTs retained RNase H catalytic activity, we measured the polymerase-dependent RNase H cleavage by wild type and mutant RTs in the absence of a trap. As shown in Figure 2, within a 5-min reaction time, wild type RT made the expected 102- and 94-nt products. Unlike the previous challenged RNase H reactions (Figure 1), N255D, N265D, and Dbl mutant RTs were able to make comparable amounts of polymerase-dependent RNase H cleavage products (Figure 2). Both the overall amounts and size distribution of cleavage products were similar between wild type and mutant RTs under these conditions. Thus, the aptamer-resistance mutations do affect RNase H under conditions that require re-binding.

## Discussion

Our results highlight several key features of the aptamer-resistant RTs bearing the mutations N255D, N265D or both (Dbl). First, each mutant displayed cross-resistance to three of the 7 anti-RT aptamers tested (Table 1). Interestingly, with three of the aptamers (RT26, RT4 and RT6), the pattern of resistance was very similar to that seen with RT1t49 in that the reduction in susceptibility was small in the case of RTs containing single mutations, and it was greater for the Dbl mutant. As shown previously, the level of resistance of each of the RTs to RT1t49 directly correlated with the dissociation constants for this aptamer. In the absence of changes in affinity to normal template·primer substrate, this suggests that the affinity of the aptamer to the RT determines the degree of inhibition achieved [25]. Therefore, our results indicate that N255 and N265 are important contact points by which HIV-1 RT interacts with each of these aptamers. In earlier work, Schneider et al. [23] classified the 30 different DNA aptamers they obtained by SELEX into six families based on primary sequence and the presence of specific secondary structures (e.g., stems, loops etc.) [23]. In spite of the dissimilarity in primary and secondary structures of the different RT-binding aptamers, it is thought that they all generate very similar 3-dimensional structures allowing them to interact with a similar binding surface on the RT protein. Additional evidence in support of this is the presence of the characteristic interrupted helices present in all RT-binding aptamers. The observation that N255D and N265D mutations confer resistance to aptamers in multiple classes suggests that these aptamers all bind HIV-1 RT in a similar manner.

The cross-resistance patterns suggest some distinct differences among the anti-RT aptamers. For example, the lack of change in sensitivity of N265D mutant to aptamer RT8 (Table 1) suggests that the residue N265 may not play a key role in binding to RT8. It is also interesting that N255D mutant displays a 10-fold hypersensitivity to RT8. We surmise that N255 residue may be involved in binding to RT8 – however, abrogation of this interaction by the N255D substitution may result in a conformational change in the RT8 or RT, which may lead to better interaction with another part of RT thus increasing its affinity to the mutant RT. Our previous work shows that changes in sensitivity to inhibition by aptamers for N255D and N265D mutant RTs directly correlate with their binding affinities to the aptamer [25]. A similar 10-fold hypersensitivity of Dbl mutant to RT8 appears to reflect the observation that the effect of N255D is dominant over that of N265D in the context of both mutations.

A long-term goal of testing anti-RT aptamers is to develop them as anti-HIV agents to be administered to individuals who have drug failure due to chronic anti-retroviral treatment or for those under supervised treatment interruption [10]. Thus, it is highly desirable that aptamers are able to suppress even drug resistant viruses. Clinically relevant aptamers can be introduced via gene therapy into hematopoietic cells of HIV-infected patients undergoing antiviral therapy. Therefore, these anti-HIV aptamers will be expressed intracellularly as RNA. In this report, we have used a DNA aptamer (RT1t49) as a model to test this notion. Our results show that most NRTI-resistant RTs display only mild resistance to aptamers (1 to 2-fold) (Table 3). However, both E89G [31], which rarely occurs among clinical isolates as a primary mutation and the more commonly encountered K65R, both display a modest level of resistance to RT1t49 (3- to 5-fold). However, both of these mutant enzymes have been shown to have altered properties with respect to their interaction with template·primer. The K65R and E89G mutants have been reported to display reductions of 50% and 32% in their dissociation constants [[32,33],196,215]. Therefore, it is likely that the increased IC_50 _of these enzymes to inhibition by the aptamer RT1t49 is an indirect result of their decreased dissociation from template·primer. The results of RT1t49 susceptibility testing (Table 3) with the ddI/ddC-resistant L74V, 3TC-resistant M184V and the AZT-resistant T215Y/M41L RTs are in agreement with our previously published efficacy tests using Jurkat T cell lines expressing each of the three selected anti-RT RNA aptamers, in which all the RNA aptamers were able to efficiently suppress replication of drug-resistant HIV [19].

Testing the wild type and the aptamer-resistant mutants of HIV-1 RT for inhibition by a variety of NRTIs and NNRTIs revealed that even if aptamer-resistance were to arise in vivo, such viruses can be efficiently suppressed by conventional antiretrovirals (Table 2). These results would be relevant to a scenario when aptamers are to be administered to HIV-infected individuals, possibly via hematopoietic stem cell therapy followed by bone marrow transplantation. In the event that aptamer-resistant variants would arise in such patients, standard RTIs can still be used to treat such patients.

The above observation, however, was tempered by the fact that some of the NRTI-resistance mutations, such as E89G and K65R conferred a significant degree of resistance to RT1t49 (3 to 5-fold). On the one hand, these results suggest that pre-existing NRTI-resistance mutations, due to altered affinities to template·primer can confer co-resistance to aptamers or that mutations such as K65R could arise in response to aptamer therapy. On the other hand, the resistance data provides insights into indirect means by which aptamer-RT interactions can be altered. Aptamer resistance can result from either a direct disruption of contact of the mutated residue with the aptamer or from an indirect effect on the conformation of a neighboring amino acid residue, increasing the template·primer affinity thus indirectly leading to altered susceptibility to the aptamer.

Although resistance to aptamers can be generated by specific mutations, our earlier work shows that these mutations alone reduce the virus infectivity by 12- to 30-fold over wild type in a single round of infection using an LTR-*lacZ *reporter cell line [25]. In addition, during a multi-day replication experiment using CD4 T cells in culture, all three viruses were unable to replicate and spread through the culture [25]. Both N255 and N265 are adjacent to the residues that form the MGBT of HIV-1 RT. The MGBT has been shown to be critical for translocation of the enzyme along the template·primer during polymerization [27]. In addition, as shown by our earlier studies, both N255D and N265D mutations affected the DNA-dependent DNA polymerase processivity, while N255D was also defective for RNA-dependent DNA polymerase processivity [26]. Our current results show that while the gross RNAse H activity is unaffected under conditions that allow re-binding (Figure 2), the processive RNAse H activity (under conditions that prevent re-binding) is affected for all three mutants (Figure 1). Thus, these mutations appear to diminish the ability of HIV-1 RT to associate with and utilize its nucleic acid substrate, therefore resulting in multiple functional defects that contribute to loss of replication fitness for the aptamer-resistant viruses. We believe that this may help explain our inability to select for resistant variants using cell lines expressing RNA aptamers (P. Joshi and V. Prasad, unpublished observations).

## Conclusion

The results presented in this report attempt to unravel the wider significance of the only two mutations previously known to specifically alter sensitivity to anti-HIV-1 RT aptamers. The mutations N255D and N265D both conferred resistance to two of the 5 new DNA aptamers (with the exception of RT8) and 1 RNA aptamer tested suggesting that the N255 and N265 residues probably serve as contact points for most aptamers. Thus, it is likely that selection with the other aptamers may also lead to these same mutations. Interestingly, the mutations N255D or N265D do not affect sensitivity to any of the NRTIs or NNRTIs tested which is a useful feature if the same mutations were to arise in response to treatment with anti-RT aptamer RNAs via gene therapy in the future. Previous results showed that these two mutations, when reconstituted into molecular clones of HIV, lead to replication defective viruses. The effects documented here, on RNase H function, combined with defects in the processive synthesis of DNA previously shown, provide additional rationale for the loss of replication competence for such viruses.

## Methods

### Polymerization assays

#### Sensitivity to inhibition by aptamers, NRTIs and NNRTIs

The sensitivity of wild type and mutant RTs to DNA and RNA aptamers, NRTIs and NNRTIs was measured in standard RT reactions essentially as described earlier [34] with the exception that 16S rRNA (Roche Diagnostics, Indianapolis, Indiana) annealed to VP200 (5'-TAACCTTGCGGCCGTACTCCCC-3') was used as template·primer. Reaction mixtures (50 μl) contained 24 nM template·primer, 80 mM KCl, 50 mM Tris-Cl (pH 8.0), 6 mM MgCl_2_, 1 mM dithiothreitol (DTT), 0.1 mg/ml BSA, 10 μM [α-^32^P] dGTP or TTP, 25 μM each of the remaining three dNTPs and a range of concentrations of DNA and RNA aptamers. Reactions, initiated by the addition of 25ng of each RT (corresponding to 10, 79, 16 and 28 units respectively for wild type, N255D, N265D and Dbl) were incubated at 37°C for 15 min. IC_50 _values of each inhibitor for a given RT variant were determined by fitting results from at least three independent experiments to a dose-response curve using nonlinear regression (GraphPad Software Inc., San Diego) using the following equation:



#### RNase H Assays

##### Challenged, polymerase-dependent and RNA 5'-end-directed cleavages

To measure the ability of enzymes to cleave RNA:DNA duplexes as the result of a single binding event, a heparin trap was added to bind any unbound enzyme or enzyme dissociated from the duplex following cleavage. Polymerase-dependent reactions included a 30-nt DNA primer annealed to a 142-nt RNA template [35]. For RNA 5'-end-directed reactions, a 41-nt RNA primer was annealed to a 47-nt DNA template. In both cases, RNA was 5'-end labeled using [γ-32P]ATP (3000 Ci/mmol) in the presence of T4 polynucleotide kinase. Final reaction mixtures (25 μl) contained 25 mM Tris-HCl (pH 8.0), 1 mM DTT, 34 mM KCl, 6 mM MgCl2, 0.5 mM EDTA, 4 nM substrate, 4 mg/ml heparin, and 0.85 nM. The reactions were initiated with MgCl2, incubated for 15 min at 37°C, and then terminated with 25 μl stop solution. Polymerase-dependent and RNA 5'-end-directed cleavage products were resolved using denaturing 6 and 12% PAGE, respectively followed by phosphorimager analysis. Control reactions were carried out using an RNase H-defective mutant of RT, E478Q [36] showing no cleavage of the RNA:DNA duplex

#### Unchallenged, polymerase-dependent cleavages

Similar to challenged reactions, for unchallenged polymerase-dependent RNAse H reactions, a 30-nt DNA primer was annealed to a 142-nt RNA template [35]. The RNA template was 5'-end labelled using [γ-^32^P]ATP (3000 Ci/mmol) in the presence of T4 polynucleotide kinase. Reactions (100 μl) were performed under the following conditions: 3.4 nM RT, 4 nM 5'- [^32^P]-labeled 142-nt RNA template annealed to a 30-nt DNA primer, 25 mM Tris-HCl (pH 8.0), 1 mM DTT, 34 mM KCl, 6 mM MgCl_2_, and 0.5 mM EDTA. RT was preincubated with the RNA:DNA substrate in the absence of MgCl_2 _for 5 min at 37°C. Reactions were initiated by the addition of MgCl_2_, and at various time points (0, 30s, 60s, 120s) an aliquot (25 μl) was removed and combined with 25 μl stop solution to stop cleavage. Cleavage products were analyzed by denaturing 6% PAGE. RNase H-directed cleavage was detected by drying the gels followed by phosphorimager analysis.

## Competing interests

The author(s) declare that they have no competing interests.

## Authors' contributions

TF carried out the drug sensitivity studies for all the aptamers using recombinant purified wild type and mutant RTs that he previously purified, performed RNase H assays and prepared the initial draft of the manuscript. PJ carried out RT inhibition studies with all the NRTIs and NNRTIs. VP conceived of the study, participated in its design and coordination and helped to generate the final manuscript. All authors read and approved the final manuscript.
